# CYP2B6 genetic variation in cyclophosphamide metabolism and hemorrhagic cystitis in Fanconi anemia patients undergoing allogeneic hematopoietic cell transplantation: A descriptive genetic association study

**DOI:** 10.1097/MD.0000000000041937

**Published:** 2025-03-21

**Authors:** Asmaa Ferdjallah, Susie Long, Todd E. DeFor, Cody Hoffmann, John E. Wagner, Pamala Jacobson, Margaret L. MacMillan

**Affiliations:** a Blood and Marrow Transplant Program, University of Minnesota, Minneapolis, MN; b Department of Pediatrics, University of Minnesota, Minneapolis, MN; c Biostatistics and Informatics Core Masonic Cancer Center, University of Minnesota, Minneapolis, MN; d Genomics Center, University of Minnesota, Minneapolis, MN; e Department of Experimental and Clinical Pharmacology, College of Pharmacy, University of Minnesota, Minneapolis, MN.

**Keywords:** Fanconi anemia, pediatrics, pharmacogenomics, stem cell transplant

## Abstract

Fanconi anemia (FA) is an inherited disorder characterized by congenital malformations, bone marrow failure, and malignancies. Hematopoietic cell transplant (HCT) is the only proven cure for the hematological complications. FA patients have increased chromosomal instability and aberrant deoxyribonucleic acid repair and thus can only tolerate low doses of chemotherapy or radiation as part of conditioning prior to HCT. Yet, they are still prone to severe regimen related toxicities including hemorrhagic cystitis (HC) from cyclophosphamide (CY). As CYP2B6 is a primary enzyme responsible for the catalyzation of the prodrug form of CY, understanding the association between CYP2B6 genetic variants and HC in FA patients may predict which patients will be more susceptible to developing HC. A descriptive genetic association study was performed to identify genetic variants associated with HC in patients with FA who underwent HCT between 1999 and 2017. All patients received a CY-based preparative regimen and had pretransplant recipient deoxyribonucleic acid available for genomic analysis. Forty FA pediatric patients were eligible for this analysis. They had received HCT from matched sibling donors (n = 6) or alternative donors (n = 34) for marrow failure (n = 38) or myelodysplastic syndrome (n = 2). The incidence of HC was 32.5% which occurred at a median of 32 days (range 20–180) after HCT. 9 patients had a concomitant viral infection (BK virus, n = 8 both adenovirus and BK virus, n = 1). No genetic variants were significantly associated with HC. The top variants were rs2279343 (g.23060A > G), and rs2279344 (g.23280G > A) in the CYP2B6 gene. The incidence of HC among FA patients with the rs2279343 variant was 42% (CI 22%–62%) compared to 20% (CI 0%–40%) among those without the variant (*P* = .19). The incidence of HC among patients with the variant in rs2279344 was 40% (CI 22%–58%) compared to 10% (CI 0%–28%) among those without (*P* = .11). No variants in our analysis were statistically associated with HC. The data suggest that CYP2B6 variants may increase the risk for HC in FA patients who received a CY based preparative therapy but these risk variants must be further evaluated in a larger population.

## 1. Introduction

Fanconi anemia (FA) is an inherited disorder marked by congenital malformations, progressive bone marrow failure, and predisposition to acute myeloid leukemia and solid tumors.^[1,2]^ It is a rare genetic disease affecting 1 in 130,000 births for which hematopoietic cell transplant (HCT) is the only cure for the hematological complications.^[3]^ Patients with FA have increased chromosomal instability and aberrant deoxyribonucleic acid (DNA) repair mechanisms, including homologous recombination, DNA mismatch repair, nucleotide excision repair, and translesion DNA synthesis.^[4,5]^ Additionally, in vitro studies confirm increased sensitivity of cells in FA patients to alkylating agents.^[6]^ Therefore, optimization of HCT conditioning regimens to achieve engraftment with minimal toxicities is extraordinarily challenging.

Cyclophosphamide (CY) an alkylating agent induces chromosomal aberrations and DNA damage in human cells, and is commonly used in the conditioning regimen for FA patients.^[6,7]^ Severe hemorrhagic cystitis (HC) is a potentially debilitating complication of CY administration and occurs in 20%–50% of FA patients after HCT.^[8,9]^ Known risk factors for HC include higher single and cumulative CY doses and its use in conjunction with a myeloablative conditioning regimen prior to HCT.^[10]^ Patients who develop viral infections with BK, adenovirus, or cytomegalovirus may also be more likely to develop HC.^[11,12]^ Although these specific risk factors are known, their role in HC is not very predictive.

CY has a complex metabolic pathway with many proteins involved in its biotransformation. After intravenous administration, CY, a prodrug, is rapidly metabolized by the liver in a multistep process to its principal active alkylating metabolite phosphoramide mustard and its inactive nonalkylating metabolite acrolein.^[13–16]^ CYP2B6, CYP2C19, CYP3A4, and CYP2C9 are the primary enzymes responsible for the initial step in bioactivation of CY.^[17]^ In particular, CYP2B6 gene is highly polymorphic with over 100 known variants and responsible for the bioactivation of CY to 4-hydroxycyclophosphamide and then to aldophosphamide which ultimately forms acrolein, the bladder toxic metabolite.^[18–20]^ Variability in the activity of the CYP2B6 enzyme is thought to explain the high degree of inter-patient variation in its pharmacokinetics of CY.^[21,22]^ Importantly, CYP2B6 variants are responsible for the varying metabolism of many clinically relevant drugs including methadone, imatinib, and efavirenz among others.^[23,24]^

HC is a common complication after HCT in FA patients receiving CY and may cause significant morbidity. We hypothesized that genetic variants in the CYP2B6 gene which encodes for CYP2B6 enzyme may result in variability of the formation of acrolein and contribute to the high rate of HC observed in FA patients. Biomarkers for the development of HC have not yet been identified but they would be helpful in selecting a preparative regimen for FA patients. The objective of this study was to evaluate the association of CYP2B6 genetic variants with HC.

## 2. Methods

### 2.1. Study population

This is a retrospective, single center cohort study of 40 pediatric patients with FA. Inclusion criteria consisted of FA patients between the ages of 0 to 21 who underwent an Institutional review board-approved allogeneic HCT at the University of Minnesota between 1999 and 2017, who consented to storing samples for future research and had serum DNA samples available for genomic analysis.

### 2.2. CY administration

All patients received CY intravenously (IV) as part of their conditioning regimen as previously described.^[25–37]^ 5 patients underwent a non-TBI containing regimen and received CY 5 mg/kg/day, IV (days -6 to -3 of HSCT) for a cumulative CY dose of 20 mg/kg. CY 5 mg/kg was administered as a 2-hour infusion with strict attention to vigorous hydration, fluid balance and maintenance of good urine output. The remaining 35 patients received a TBI containing regimen with IV CY 10 mg/kg/day (days -5 to days -2) for a cumulative CY dose of 40 mg/kg. All patients received IV mesna (total 60 mg/kg/day in 5 divided doses) prior to CY, and 3, 6, 9, and 12 hours after CY to reduce the risk of HC. Adequate diuresis was maintained before and after CY administration to help prevent HC. All patients received hyperhydration with IV fluids at 3000 cc/m^2^/24 hours initiated at least 6 hours before the CY dose. Furosemide IV was administered as needed to maintain high urine output and frequent voiding (at least q2 hours) to decrease toxic acrolein exposure. Additionally, patients were weighed twice daily from day -2 to day of HCT to guide diuresis.

Patient and transplant characteristics are shown in Table 1. The median patient age at diagnosis was 8 years (range 5–21). 38 patients had severe marrow failure and 2 had myelodysplastic syndrome. Graft sources included human leukocyte antigen-identical sibling bone marrow (BM; n = 6) a 1 antigen mismatched related BM (n = 5), matched unrelated donor (URD) BM (n = 19), 1 antigen mismatched URD BM (n = 2), and single (n = 7) or double (n = 1) umbilical cord blood.

**Table 1:** Participant characteristics; CY: cyclophosphamide, CY(20); cyclophosphamide 20 mg/kg cumulative dose, CY(40) cyclophosphamide 40 mg/kg cumulative dose, sUCB: single umbilical cord blood, dUCB: double umbilical cord blood, HCT: hematopoietic stem cell transplant, URD: unrelated donor.

Characteristic	Frequency (n, %)
N	40
Age at HCT	
Median (range)	8 (5–21)
Gender: Male	20 (50%)
Donor source	
Matched sibling	6 (15%)
7/8 HLA mismatched related BM	5 (13%)
8/8 HLA matched unrelated BM	19 (48%)
7/8 HLA mismatched unrelated BM	2 (5%)
sUCB	7 (18%)
dUCB	1 (3%)
Conditioning regimen (total CY dose in mg/kg)	
TBI, CY(40), Flu	21 (65%)
TBI, CY(40), Flu, ATG	9 (23%)
TBI, CY(40), ATG	2 (5%)
• TBI, CY(20), Flu	1 (3%)
CY(20), Flu, ATG	5 (13%)
CY(40), Flu, Bu, ATG	2 (5%)

### 2.3. Data collection and toxicity evaluation criteria

Clinical characteristics and HC data was obtained from the University of Minnesota Bone Marrow Transplant Database which uniformly collects and aggregates transplant data. This study was Institutional review board-approved and all parents/guardians signed informed consents in accordance with the Declaration of Helsinki.

HC was graded on a 0 to 4 scale according to the grading system developed by Droller et al as shown in Table 2.^[38]^ Patients were assessed daily while inpatient and at each clinic appointment while outpatient for HC and upon report of dysuria, each patient underwent a urinalysis to screen for hematuria. Grading was performed by the treating physician at the time of clinical diagnosis of HC and subsequently independently confirmed by a study investigator upon chart review. Each patient was followed until resolution of HC and none had recurrence.

**Table 2:** Grading of hemorrhagic cystitis toxicity.^[38]^

Grade	Symptoms
0	No symptoms of bladder irritability or hemorrhage
1	Microscopic hematuria
2	Macroscopic hematuria
3	Macroscopic hematuria with small clots
4	Massive macroscopic hematuria requiring instrumentation for clot evacuation and/or causing urinary obstruction

### 2.4. Genetic variant selection and genotyping

Genetic variants in the CYP2B6 gene, which encode for proteins involved in the CY metabolic pathway and formation of acrolein, were identified through a review of the published literature and 11 variants were selected for evaluation (Table 3). Previously stored recipient DNA isolated from blood obtained prior to the initiation of conditioning therapy was analyzed for the variants.

**Table 3:** Candidate variants and haplotype evaluated and frequencies for the known effect; global prevalance.^[23]^

Variant*	Risk allele (for known effect)	Frequency	Known effect
rs4802101 g.4258T > A/C/G	C	8/10	↑ Metabolism of 4-OH-CY and risk of toxicity^[39]^
rs8192719 g.26570C > T	T	6/10	↓ Metabolizing activity of CYP2B6 enzyme^[40]^
rs4803418 g.19600C > G	G	1/3	↓ Metabolizing activity of CYP2B6 enzyme^[41]^
rs2279344 g.23280G > A	A	¾	↓ Metabolizing activity of CYP2B6 enzyme^[42]^
rs28399499 g.26018T > C	C	0	↓ Metabolizing activity of CYP2B6 enzyme^[43]^
rs7254579 g.2688T > C	C	39/100	↑ Cy metabolites^[44]^
rs8192709 g.5071C > T	T	0	↑ Hemorrhagic cystitis
rs2279343 g.23060A > G	G	62/100	↓ Cy clearance^[45]^
rs3211371 g.30512C > A/T	T	25/100	↓ Cy efficacy^[46]^ ↓ Cy toxicity^[46]^
rs3745274 g.20638G > T and rs2279343 g.23060A > G (together *6)	T, G	0	↑ VOD^[21]^
rs12721655 g.18079A > G	G	0	↓ Metabolizing activity of CYP2B6 enzyme^[42]^

* CYP2B6 RefSeqGene (LRG_1267).

Genotyping was performed using the iPLEX Gold method. iPLEX reagents and protocols for the multiplex polymerase chain reaction (PCR), single base primer extension and generation of mass spectra, as per the manufacturer’s instructions (for complete details see iPLEX Application Note, Agena, San Diego, CA) were utilized. qPCR was performed in 5-µL reactions on 384-well plates containing 10 ng of dried down genomic DNA. Reactions contained 2X TaqMan Universal PCR Master Mix (Applied Biosystems, 20X primer and Taqman primer-probe mix for RNAse P with VIC dye). Following enzyme activation at 95 °C for 10 min, DNA was amplified with 40 cycles of 92 °C × 15 sec and 60 °C × 1 min. Amplification was performed and measured on Applied Biosystems QuantStudio 7 Real-Time PCR System (Applied Biosystems, Foster City, CA). Amplification analysis was performed on the QuantStudio Design and Analysis software (Applied Biosystems, Foster City, CA).^[47]^ All variants were in Hardy-Weinberg equilibrium.^[48]^

### 2.5. Statistical analysis

Given the sample size and the small number of events, assessment of risk of HC by candidate genomic variants was performed by a descriptive hypothesis generating univariate analysis of candidate variants in Table 2 along with CY (20 mg/kg vs 40 mg/kg), donor type (sibling vs mismatched related vs URD vs UCB), year of transplant (1995–2010 vs 2011–2018) and cytomegalovirus serostatus (negative vs positive). The primary endpoint of HC by 6 months after HCT was estimated using cumulative incidence treating non-cystitis death as a competing risk along with 95% confidence intervals. Only 2 patients died among this cohort at 1.7- and 15-years after HCT. Gray’s test was used to complete comparisons by genomic variants.^[39]^ Patients who were heterozygous or homozygous for the risk allele were combined together and compared to those without the allele. *P*-values were two-sided. Analyses were performed using statistical analysis system 9.4 (SAS Institute, Inc., Cary, NC).

## 3. Results

### 3.1. Patient and transplant characteristics and outcomes

Patient and transplant characteristics are shown in Table 1. Median (range) time to neutrophil and platelet engraftment was 13 days (9–45) and 28 days (15–87), respectively.

Of the 40 patients in this cohort, 13 (32.5%) patients developed HC at a median of 32 days (range, 20–180, interquartile range 23–36 days) after HCT. Severity of HC was grade 1 (n = 3), grade 2 (n = 5), grade 3 (n = 4), and grade 4 HC (n = 1). Duration of HC did not correlate with severity. 5 patients experienced mild symptoms not necessitating intervention. Interventions administered included hyperhydration (n = 5), phenazopyridine (n = 5), and oxybutynin (n = 4). Pain management utilized included morphine (n = 2), oxycodone (n = 4), and lidocaine (n = 2). 2 patients received cidofovir. The most common isolated viral infection was BK virus (n = 5). A single patient had BK viruria without viremia. Only 1 patient tested positive for adenovirus viremia while no patients had adenovirus viruria.

### 3.2. HC and CYP2B6 genetic variants

The presence and frequency of the CYP2B6 variant by presence of HC is shown in Table 4. Of the 13 patients who developed HC, the most frequently identified variant was rs2279344 (n = 18, 69.2%). Of the 27 patients who did not develop HC, the most frequently identified variant was rs4802101 (n = 32, 59% of patients).

**Table 4:** Presence of the variants in FA patients with and without hemorrhagic cystitis.

CYP2B6 variants	Variant (frequency), n (%) in FA patients without HC (n = 27)	Variant (frequency), n (%) in FA patients with HC (n = 13)
rs4802101	32 (59%)	17 (65%)
rs8192719	17 (31%)	11 (42%)
rs4803418	8 (15%)	6 (23%)
rs2279344	28 (52%)	18 (69%)
rs28399499	0 (0%)	0 (0%)
rs7254579*	9 (18%)	7 (27%)
rs8192709	0 (0%)	0 (0%)
rs2279343†	17 (33%)	13 (50%)
rs3211371	6 (11%)	4 (15%)
Both rs3745274 & rs2279343	0 (0%)‡	1 (8%)
rs12721655	0 (0%)	0 (0%)

* Variant data not available for 2 patients; both of which did not have hemorrhagic cystitis; n = 50.

† Variant data not available for 1 patient; who did not have hemorrhagic cystitis; n = 52.

‡ Complete variant data not available for 6 patients; none of which had hemorrhagic cystitis; n = 42.

Among FA patients, none of the tested alleles were associated with HC (Table 5). The 2 variants, rs2279343 and rs2279344, showed a trend towards an association (*P* = .19 and *P* = .11, respectively). *P*-values for other variants were > .25.

**Table 5:** Association of hemorrhagic cystitis and genetic variants through 6 months.

rs number (risk allele)	Presence of risk allele	HC frequency			HC cumulative incidence		*P*-value
		N	Events	Median days to HC (range)	Estimate	CI 95	
rs2279343 (G)	No	15	3	25 (23–34)	20%	0%–40%	.19
	Yes	24	10	32 (20–180)	42%	22%–62%	
rs2279344 (A)	No	10	1	21	10%	0%–28%	.11
	Yes	30	12	32 (20–180)	40%	22%–58%	
rs3211371 (T)	No	30	9	32 (20–180)	30%	14%–46%	.50
	Yes	10	4	24 (21–51)	40%	10%–70%	
rs4802101 (C)	No	8	2	24 (23–25)	25%	0%–53%	.68
	Yes	32	11	32 (20–180)	34%	18%–51%	
rs4803418 (G)	No	27	7	25 (20–36)	26%	10%–42%	.27
	Yes	13	6	41.5 (21–180)	46%	19%–73%	
rs7254579 (C)	No	23	6	27.5 (20–36)	26%	8%–44%	.25
	Yes	15	7	32 (21–180)	47%	21%–72%	
rs8192719 (T)	No	16	4	37 (21–93)	25%	4%–46%	.40
	Yes	24	9	32 (20–180)	38%	18%–57%	
rs3745274 (T)	No	12	3	51 (23–93)	25%	1%–49%	.37
	Yes	23	9	32 (20–180)	39%	19%–59%	

**Figure 1.**
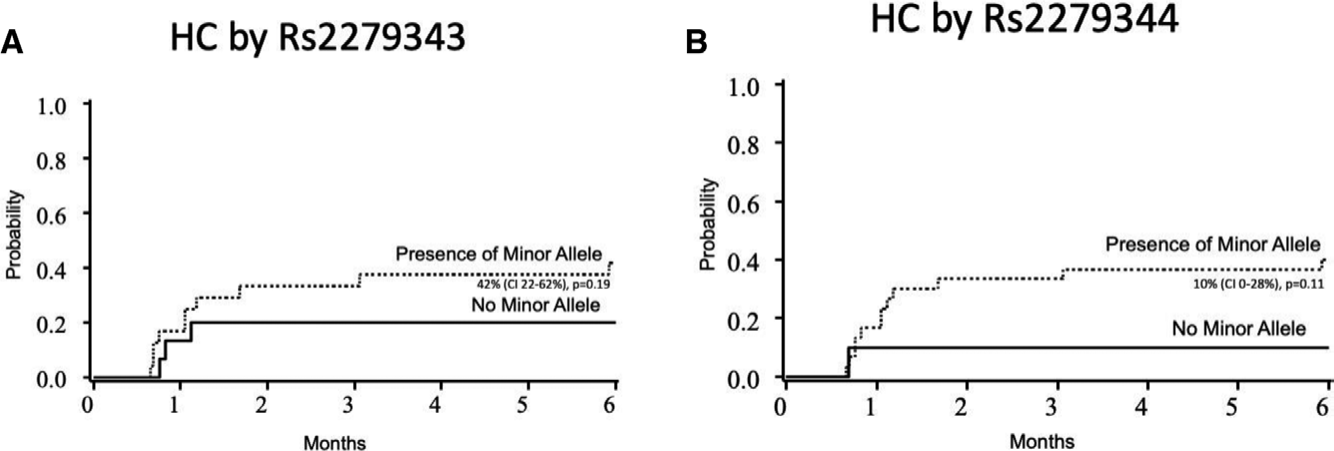
(A) The probability of hemorrhagic cystitis over time in FA patients with the variants rs2279343 and (B) rs2279344.

Of the 15 patients with rs2279343, 42% (CI 22%–62%) developed HC compared to 20% (CI 0%–40%) in those without the variant (*P* = .19). Of the 10 patients with rs2279344, 40% developed HC (CI 22%–58%) compared to 10% (CI 0%–28%) in those without this variant (*P* = .11). Figure 1 highlights the probability of HC over time in FA patients with the variants rs2279343 and rs2279344.

Other potential confounders including CY dose (20 or 40 mg/kg), donor type, year of transplant (1995–2010 or 2011–2018) and cytomegalovirus serostatus were not significantly associated with the development of HC.

## 4. Discussion

In our study, we report the effect of CYP2B6 variants on the incidence of HC in a cohort of FA patients undergoing HCT. Due to the small cohort, all grades of HC were included. We observed a higher frequency of HC among FA patients who carried the CYP2B6 variants rs2279343 and rs2279344. Rs2279343 is CYP2B6*4 and is found in all major ethnic groups, particularly in approximately 50% of Black or African Americans.^[40]^ In our cohort, none of the FA patients who developed HC were Black and the only Black patient (who did not develop HC) carried the rs2279343 variant. Rs2279343 has been associated with a higher CYP2B6 protein expression in several studies and increased functional status of the enzyme with a variety of drugs in vivo and in vitro.^[41]^ Rocha et al reported in 107 patients with leukemia, that rs2279343 was associated with an increase in moderate to very severe mucositis (*P* = .007) with an odds ratio of 3.03 but no association with HC.^[42]^ Ariyoshi et al reported that CY metabolism is decreased in the presence of the rs2279343 variant in vivo.^[43]^ In our cohort, we hypothesize that the presence of the rs2279343 variant increases the functional status of the CYP2B6 enzyme and more acrolein is subsequently formed as a downstream byproduct leading to bladder toxicity.

The variant rs2279344 has not been studied extensively and little data exists, particularly as it relates to CY metabolism. Current studies discuss the variant in methadone metabolism.^[44]^ Although Dobrinas et al report that the variant is associated with increased CYP2B6 activity, Ahmad et al report that the variant is associated with decreased expression or enzyme activity of CYP2B6.^[45]^ More studies are needed to determine true directionality of the variant. Like rs2279343, the rs2279344 variant frequency is higher among FA patients who develop HC although with a *P*-value not significant but suggestive.

Overall, our findings indicate a role for pre-allogeneic stem cell transplant pharmacogenomic studies in FA patients. Identifying the presence of these variants can guide practitioners in adequate prevention of HC. Simple measures such as hyperhydration and anti-bladder spasmodics are benign and easy to institute. A primary limitation to this study is the sample size. Other limitations include the fact that many patients also receive both CYP inhibitors and inducers during the transplant period, including fluconazole, trimethoprim/sulfamethoxazole, and itraconazole. Other medications including steroids and antiemetics may also interfere with the autoinduction of certain CYP enzymes.^[46]^ Additionally, a majority of patients received total body irradiation which is a risk factor for HC. FA patients have an inherent DNA repair defect and as such, they receive about one-fifth the dose given to a non-FA patient. Other factors may play a key role including age and ethnicity combined with dose of CY. Future directions to better elucidate this relationship include CY and metabolite pharmacokinetic studies in patients with these variants. These results will serve to generate future hypotheses for larger studies and although the differences noted are not statistically significant due to low power, some are suggestive. As discussed above, for other medications metabolized by CYP2B6, there exist actionable pharmacogenomic dosing guidelines for clinical practice (i.e. efavirenz). Confirming these CY variants as actionable may lead to CY dosing guidelines.

This may suggest the presence of these variants may be associated with an incidence of HC that is 2 times greater than in the absence of rs2279343 and 4 times greater among patients with rs2279344.

## Acknowledgments

The authors thank the nurses, nurse coordinators, nurse practitioners, physician assistants, social workers and physicians who cared for these patients and their families. The authors especially thank the patients with Fanconi anemia and their families who have entrusted us with their care to continually strive to improve upon our work. The authors wish to acknowledge the University of Minnesota, Twin Cities Pediatric Bone Marrow Transplant division.

## Author contributions

**Conceptualization:** Asmaa Ferdjallah, Susie Long, Cody Hoffmann, John E. Wagner, Pamala Jacobson, Margaret L. MacMillan.

**Data curation:** Asmaa Ferdjallah, Margaret L. MacMillan.

**Formal analysis:** Susie Long, Todd E. DeFor, Cody Hoffmann, Pamala Jacobson, Margaret L. MacMillan.

**Funding acquisition:** Margaret L. MacMillan.

**Investigation:** Asmaa Ferdjallah, Susie Long, Pamala Jacobson, Margaret L. MacMillan.

**Methodology:** Asmaa Ferdjallah, Todd E. DeFor, Cody Hoffmann, Pamala Jacobson, Margaret L. MacMillan.

**Project administration:** Asmaa Ferdjallah, Pamala Jacobson, Margaret L. MacMillan.

**Resources:** Asmaa Ferdjallah, Margaret L. MacMillan.

**Software:** Margaret L. MacMillan.

**Supervision:** Pamala Jacobson, Margaret L. MacMillan.

**Validation:** Asmaa Ferdjallah, Susie Long, Margaret L. MacMillan.

**Visualization:** Susie Long, Margaret L. MacMillan.

**Writing – original draft:** Asmaa Ferdjallah.

**Writing – review & editing:** Asmaa Ferdjallah, Susie Long, Todd E. DeFor, Cody Hoffmann, John E. Wagner, Pamala Jacobson, Margaret L. MacMillan.
